# Supplemental greenhouse lighting increased the water use efficiency, crop growth, and cutting production in *Cannabis sativa*

**DOI:** 10.3389/fpls.2024.1371702

**Published:** 2024-06-07

**Authors:** Cristian E. Collado, Seung Jae Hwang, Ricardo Hernández

**Affiliations:** ^1^ Department of Horticultural Science, North Carolina State University, Raleigh, NC, United States; ^2^ Division of Horticultural Sciences, Institute of Agriculture & Life Sciences, Research Institute of Life Sciences, Division of Crop Sciences, Gyeongsang National University, Jinju, Republic of Korea

**Keywords:** HEMP, stock plant, propagation, water management, water reduction, crop yield, PPFD, DLI

## Abstract

The expanding cannabis production sector faces economic challenges, intensified by freshwater scarcity in the main US production areas. Greenhouse cultivation harnesses sunlight to reduce production costs, yet the impact of greenhouse light levels on crucial production components, such as plant growth, branching, and water use efficiency (WUE), remains poorly understood. This study aimed to assess the effects of combined sunlight and supplemental lighting on the crop’s main production components and leaf gas exchange of *Cannabis sativa* ‘Suver Haze’ in the vegetative stage. Within a greenhouse, LED lighting provided at intensities of ~150, 300, 500, and 700 µmol m^-2^ s^-1^ (18-hour photoperiod), combined with solar radiation, resulted in average daily light integrals of 17.9, 29.8, 39.5, and 51.8 mol m^-2^ d^-1^. Increasing light levels linearly increased biomass, leaf area, and the number of branches per plant and square meter, with respective rates of 0.26 g, 32.5 cm^2^, and 0.41 branches per mole of additional light. As anticipated, crop evapotranspiration increased by 1.8-fold with the increase in light intensity yet crop WUE improved by 1.6-fold when comparing the lowest and highest light treatments. Moreover, water requirements per unit of plant biomass decreased from 0.37 to 0.24 liters per gram when lighting increased from ~18 to 52 mol m^-2^ d^-1^, marking a 35% reduction in evapotranspiration. These results were supported by increments in leaf photosynthesis and WUE with light enhancement. Furthermore, our findings indicate that even 52 mol m^-2^ d^-1^ of supplemental lighting did not saturate any of the crop responses to light and can be economically viable for cannabis nurseries. In conclusion, light supplementation strongly enhanced photosynthesis and plant growth while increasing WUE. Additionally, a comprehensive discussion highlights the shared physiological mechanisms governing WUE in diverse plant species and their potential for water conservation under enhanced lighting conditions.

## Introduction

The expansion of both medicinal and recreational cannabis sectors within the United States is witnessing persistent growth, attributable to the ongoing legalization efforts across various states (DeCarcer et al., 2021). The cultivation of high-value cannabis crops is predominantly conducted within controlled environments, encompassing indoor sole-source-LED light facilities and greenhouses. While greenhouse cultivation harnesses solar energy to enhance cost-efficiency in production (Vezdos and Martinez, 2021), it confronts the challenge of potentially insufficient natural sunlight levels required to attain targeted crop quality and yield. Furthermore, the escalating demand for water resources poses a pressing concern, as approximately 37% of the freshwater supply in the United States is allocated to irrigation (Dieter et al., 2018). This issue is exacerbated by other factors, including population growth, agricultural demands, complexities in water infrastructure, and recurring drought conditions (GAO, 2014). Furthermore, California, a state deeply affected by water shortages, accounted for 58% of medical and recreational cannabis licenses issued in 2021 (Conway, 2023). Therefore, it is imperative to optimize cannabis production and other crops through the strategic application of supplemental lighting and water-saving approaches. Addressing these multifaceted challenges requires a comprehensive understanding of crop light and water utilization.

Research pertaining to *Cannabis sativa* cultivated under sole-source LED lighting reported a correlation between the daily light integral (DLI) and crop productivity. For instance, cannabis flower yield exhibited a linear increase in response to increasing DLI levels, up to 78 mol m^-2^ d^-1^, which represented the highest DLI investigated under short-day conditions, 12 h (Rodriguez-Morrison et al., 2021). Moreover, when subjected to long day durations, 16 h, the shoot biomass demonstrated a similar positive trend and increased even with 82 mol m^-2^ d^-1^ (Moher et al., 2021). However, it is essential to acknowledge that the dynamics of light within the context of greenhouse production can deviate substantially from those observed in sole-source LED facilities, and those changes may strongly affect plant physiological and crop efficiencies. Greenhouse environments are characterized by diurnal fluctuations in light intensity, a balanced spectrum encompassing blue, green, red, and far-red wavelengths, as well as the introduction of additional radiation from 800 to 2500 nm. The resultant but dynamic spectral combination between sunlight and electrical lighting further confounds the light environment within greenhouses. These dynamic light conditions can influence diverse facets of plant biology, including growth patterns, morphological attributes, and key physiological processes such as photosynthesis, stomatal conductance, transpiration, and energy balance (Pieruschka et al., 2010; Nelson and Bugbee, 2015; Zhen and Bugbee, 2020). Consequently, it is imperative to emphasize the need for comprehensive characterizations of the lighting environment in greenhouse supplemental lighting studies. Moreover, this research enhances the limited body of knowledge concerning the impact of supplemental lighting on cannabis greenhouse production (David Potter, 2009).

Radiation influences water usage in non-stressed plants, primarily by increasing transpiration rates (Whitehead, 1998; Pieruschka et al., 2010; Pokorny, 2019). While it is known that increased radiation levels correspondingly lead to increased water consumption, a more comprehensive approach for assessing water utilization leads to calculating water-use efficiency (WUE). WUE captures the interaction between plant growth parameters, such as yield and biomass, with transpiration or evapotranspiration and can be quantified at the leaf, plant, and crop levels (Jackson et al., 2016; Hatfield and Dold, 2019).

One of the principal strategies employed to enhance crop WUE is the deliberate reduction of water availability within the soil or substrate, a practice that significantly decreases the transpiration rate. Nonetheless, this approach most often reduces plant productivity, given the associated decrease in photosynthetic rates (Hubick and Farquhar, 1989; Lawson and Blatt, 2014; Xing et al., 2022). Remarkably, research has demonstrated that trees originating from diverse botanical families and adapted to varying geographical regions exhibit reduced leaf transpiration when subjected to elevated radiation levels in comparison to conspecifics grown under lower irradiance conditions, even while concurrently displaying heightened rates of photosynthesis (Idris et al., 2019). This suggested that leaves adapted to highlight environments may possess superior WUE compared to their shade-adapted counterparts when both leaf types are subjected to identical radiation regimes. Therefore, one of the main focuses of this investigation was to quantify leaf and crop water-use efficiencies in the context of cannabis cultivation under increased supplemental light conditions.

Cannabis nurseries and flower producers employ extended photoperiods to increase branch proliferation and plant size. This enhances cutting production in nurseries and conditions plants for higher flower yields. Therefore, enhancing biomass and branch count and minimizing cultivation time is vital for cannabis nurseries, ensuring a seamless supply of plants to cannabis flower producers. Prior investigations have yet to elucidate the impact of lighting on branch development (potential cuttings) and the associated morphological and physiological parameters of cannabis vegetative plants in greenhouses.

The primary objective of this research was to investigate the impacts of greenhouse light levels, encompassing solar radiation in conjunction with various supplemental LED light intensities (~150, 300, 500, and 700 µmol m^-2^ s^-1^), on growth parameters, the number of branches, water utilization (WU), and water-use efficiency (WUE) in a vegetative crop of *Cannabis sativa* L. Additionally, we aimed to assess the effects of these prolonged light treatments on key leaf physiological attributes, specifically photosynthesis (A), stomatal conductance (g_sw_), and transpiration (E), which are pivotal determinants of both growth and water consumption.

The study involved the examination of two hypotheses. 1) Increments in the cumulative light dosage would elicit an increase in plant growth and in the number of branches. 2) Leaves of cannabis plants cultivated under an elevated DLI would display higher WUE due to increased photosynthetic rates than their counterparts exposed to a lower DLI when leaves were evaluated under high PPFDs.

## Materials and methods

### Starting plant material and location

Rooted cuttings of *Cannabis sativa* cv Suver Haze (© Oregon CBD) served as the initial material. The cuttings underwent rooting in a greenhouse for 24 days and a photoperiod of 18 hours. The propagation was conducted by a commercial nursery in Broadway, NC, US (Ryes Greenhouses LLC). On October 9, 2020, 240 plug plants (rooted cuttings) were selected to be planted, as well as five additional and representative samples for destructive measurements. The most uniform plants were selected based on morphological similarities (mean ± SD of five representative samples: number of leaves: 3.6 ± 0.9, shoot fresh and dry mass: 1.4 ± 0.4 g and 0.26 ± 0.08 g, root dry mass: 18.1 ± 7.2 mg, and leaf area: 37.5 ± 10.5 cm^2^). The plants were evenly distributed among 12 research plots within a glass greenhouse at the NC State Horticulture Field Lab in Raleigh, NC, USA. Further details can be found in **Supplementary Figure S1** of the **Supplementary Material** section.

### Plot sensing and controlling capabilities

Each production-research plot was equipped with two LED dimmable lighting fixtures to establish and maintain specific light levels, as illustrated in **Supplementary Figure S1**. Additionally, two soil moisture sensors, two load cells, and a solenoid valve were employed to quantify water evapotranspiration and sustain optimal substrate moisture and nutrition due to the crop requirements at each plot. Furthermore, a fine-wire thermocouple and quantum sensor were utilized to address greenhouse air temperature and solar variations. In total, 72 sensors and 12 solenoids were connected to a datalogger and controller through four sensor multiplexers and a relay driver, with data logged at 5-minute intervals. Hardware specifications are detailed in the **Supplementary Material** section.

### The treatments

The day after planting, the supplemental lighting was increased from ~90 µmol m^-2^ s^-1^ to ~ 150, 300, 500, and 700 µmol m^-2^ s^-1^ (**Table 1**) and maintained for 20 days until harvest. Each light level represents the average of three plots or production areas (**Supplementary Figure S1**). However, the treatments consisted of a combination of supplemental LED and solar radiation. Details are presented in **Table 1**, and the light spectra of average sunlight, as well as the lowest and highest light treatments, are presented in **Table 2** and **Supplementary Figure S2**. The calibration and control of the lights are described in the **Supplementary Material** under the title *Supplemental Light Calibration and Control*.

**Table 1:** Summary of light sources and levels: supplemental PPFD, cumulative supplemental plus solar treatments (∑PPFD), average daily light integral (DLI), average PPFD, and light source.

Supplemental PPFD^z^	∑PPFD^y^	DLI^x^	Average PPFD^w^	Light source:^v^		∑ePAR (400-750 nm)	eDLI (400-750 nm)	PPFD ePAR
µmol m^-2^ s^-1^	mol m^-2^	mol m^-2^ d^-1^	µmol m^-2^ s^-1^	**Sun**	**LED**	mol m^-2^	mol m^-2^ d^-1^	%
151 ± 0	376 ± 49	17.9	277	48%	52%	408	19.5	92%
300 ± 2	625 ± 8-	29.8	459	38%	62%	669	31.8	94%
501 ± 2	829 ± 59	39.5	610	22%	78%	862	41.1	96%
703 ± 3	1088 ± 55	51.8	800	17%	83%	1121	53.4	97%

∑ePAR and eDLI are cumulative photons based on an extended PAR range 400 to 750 nm (Zhen et al., 2021). Means and standard deviations were calculated from the three greenhouse plots per light level. The photoperiod was maintained at 18 hours.

z Photosynthetic photon flux density from 400 to 700 nm) measured with a line quantum sensor (mean ± standard deviation).

y Measured continuously for 21 days with quantum sensors (mean ± SD). Twenty-one days include one day of LED (~90 µmol m^-2^ s^-1^) + solar radiation and 20 days of LED treatments + solar radiation.

x DLI = ∑PPFD/21 days.

w DLI/Photoperiod * time and mol conversion factors.

v Proportions between supplemental (LED) and solar radiation (Sun).

**Table 2:** Solar and LED spectrum measurements and calculations for light quality comparison.

Plant light parameter	wavelength range (nm)	Units	Greenhouse sunlight (Sun)	LED (151 PPFD)	LED (703 PPFD)	Sun + LED (144 + 151 PPFD)	Sun + LED (144 + 703 PPFD)
PPFD	400-700	µmol m^-2^ s^-1^	144	151	703	295	847
ePAR*	400-750		170	152	707	321	877
UV	300-400		5.0	0.1	0.4	5.1	5.4
Blue	400-500		34.2	27.5	121.6	61.8	155.8
Green	500-600		53.3	0.4	1.5	53.6	54.7
Red	600-700		56.5	123.1	580.4	179.6	636.9
Far Red	700-800		49.3	0.9	4.0	50.2	53.3
Blue/PPFD		%	23.8	18.2	17.3	20.9	18.4
Green/PPFD			37.0	0.2	0.2	18.2	6.5
Red/PPFD			39.3	81.5	82.5	60.9	75.2
Red/Blue			1.65	4.48	4.77	2.91	4.09
Red/Far Red			1.15	132.0	143.7	3.6	11.9
Pfr/Ptotal**			0.73	0.88	0.88	0.83	0.86

The greenhouse sunlight measurements were adjusted to match the average photosynthetic photon flux density of 144 µmol m^-2^ s^-1^. The calculated solar mean is based on an 18-h photoperiod and an average DLI of 9.3 mol m^-2^ d^-1^ from the solar radiation measurements among the 12 plots. The LED and Sun + LED columns show the lowest and highest lighting treatments. The LED columns represent the greenhouse lighting conditions without solar radiation, while the Sun + LED columns represent average lighting conditions in the 18-h lighting period. This table analysis is based on ~20 and 21 days for the LED conditions and the average greenhouse solar radiation, respectively; thereby, they may differ from values in **Table 1**.

*ePAR represents an extended PAR or PPFD range (Zhen et al., 2021).

**Phytochrome photoequilibrium (Sager et al., 1988).

### Greenhouse conditions

The greenhouse air conditions were regulated with forced hot air, a cooling wet pad, two exhaust fans, and a Priva Maximizer control system (De Lier, Netherlands). The average temperature and humidity were 25.4 ± 1.7°C and 73.3 ± 10.4% (mean ± SD) at the center of the greenhouse at canopy height and measured in an aspirated box. The air temperature at the top of the 12 canopies was similar, yielding a mean ± standard deviation between plots of 25.4 ± 0.3°C. The daytime carbon dioxide was around 410 µmol mol^-1^ and monitored right next to the canopy.

### Control of the substrate moisture and fertilization

Control of substrate moisture and fertilization was managed through independent irrigation control at each of the 12 plots, utilizing readings from two soil volumetric water content (VWC) sensors per plot. The plot’s solenoid valve was activated for 25 minutes based on the lowest substrate moisture reading from two plants in each treatment area. Irrigation was initiated when the VWC reached or fell below 0.345 ± 0.014 m^3^ m^-3^ (80% of container capacity).

Two irrigation emitters with a combined flow of 5.4 L h^-1^ per pot were employed to ensure uniform moisture and prevent nutrient-salt accumulation. Detailed analysis of nutrient solution composition, pour-thru procedure samples (**Table S1**), substrate conditions, and moisture calibration can be found in the **Supplementary Material** section.

### Plants for measurements and growing area

the total growing area per plot was made of 20 plants, using a density of 10.8 plants m^-2^. However, only plants from the center of the plots (6 plants) were used for plant measurements to avoid edge effects.

### Vegetative growth measurements

Plant height (measured from the growing tip to substrate level) was assessed every two to four days. Plant biomass and leaf parameters were determined through destructive measurements on day 21. Plant fresh mass was immediately recorded after cutting the plant at ground level. Leaf area, and leaf and stem dry mass were quantified using Li-3100C units (Li-Cor, Lincoln, NE, USA) and drying ovens set at 69°C, respectively. The number of primary and secondary branches (length ≥ 5 cm) was counted on the main stem and the primary branches, respectively.

### Evapotranspiration

Evapotranspiration (ET) and evaporation were quantified by measuring changes in the load cell readings (including the weight of water, substrate, pot, and plant for ET, or the weight of water, substrate, and pot for evaporation) after and immediately before the irrigation events occurred (**Supplementary Figure S3A**). ET was quantified in all plots, while evaporation was measured using a pot without a plant. Refer to the **Supplementary Material** section for more details.

WUE (Water Use Efficiency): Shoot water use efficiency was calculated as the ratio between the dry mass of leaves and stems and the litters of evapotranspiration in a 21-day period. Similarly, the ratios between primary, secondary, and total branches and evapotranspiration requirements were calculated. Leaf WUE was calculated as the ratio between photosynthesis (A) and transpiration (E).

### Metabolite and gas exchange measurements

These measurements were performed on fully expanded, light-exposed top leaves to compare leaves developed under different light levels. Crops were subjected to light treatments for 18 days before the readings. Chlorophyll, anthocyanin, and flavonol indexes were measured on the adaxial surfaces using a Dualex Scientific handheld leaf-clip meter (Force-A, Orsay, France). On similar leaves, net CO_2_ assimilation (A), stomatal conductance to water vapor (g_sw_), transpiration (E), internal leaf CO_2_ concentration (C_i_), and leaf temperature measurements were taken using a Li-6800 with a 2-cm² aperture 6800-01A chamber (Li-Cor, Lincoln, NE, USA). Readings were recorded at the center of the leaflets and after the CO_2_ assimilation plateaued for at least two minutes. The chamber maintained 18% blue light, 82% red light, 70% relative humidity (RH), 27°C temperature, and 404 ppm of CO_2_. The settings were chosen based on greenhouse conditions and instrument capability.

### Statistics

For crop parameters and leaf pigments, linear regression and ANOVA were conducted using the mean of one or more subsamples per plot. Subsamples or observational units (OU) consisted of 6 plants for plant height, 3 plants for biomass, leaf area, and branches, 1-2 load cells for evapotranspiration, and 18 leaves for metabolites. The total sample size was defined as N, and in the same way, the number of true replications in one group or light level was n. Because the plot acted as the experimental unit, N and the number of plots were equivalent to 12 (Lazic et al., 2018).

For leaf gas exchange analysis from 150 to 2000 µmol of light m^-2^ s^-1^ (PPFD), only top leaves grown under DLIs of ~18 and 52 mol m^-2^ d^-1^ were analyzed (N=6, n=3, OU=1 leaf per plot). Split-plot models were used on A, E, g_sw_, and WUE to evaluate responses based on the effects of DLI, PPFD, and DLI*PPFD. Light intensity (150, 300, 500, 700, 1000, 1300, 1600, and 2000 µmol m⁻² s⁻¹) effects were evaluated at the split-plot level. Consequently, the effects of long light exposure or DLI were assessed at the main plot level. In addition, DLI effects were analyzed using t-tests. T-tests were used to compare specific groups and validate this study’s second hypothesis. More specifically, one-tailed t-tests were used to evaluate the effect of DLI on leaf photosynthesis and WUE under elevated PPFDs (≥ 700 µmol m^-2^ s^-1^), while leaves under lower PPFDs were evaluated under two-tailed t-tests. All analyses and graphs were performed using JMP Pro 16.0 and 17.0 from SAS (Cary, NC, USA).

## Results

The results were analyzed using the cumulative light per square meter exposure in 21 days (∑PPFD), given that plant responses to light are influenced by the spatial and temporal aspects of light capture (Rosati and Dejong, 2003). Nevertheless, considering that the standard practice in research and production often involves quantifying cumulative light on a daily basis rather than over the entire growth period, Daily Light Integral (DLI) was also used for the interpretation of results and discussion. A comprehensive dataset, including specific and general crop responses to light, was provided in **Table 3** to facilitate discussion. Additional results, such as leaf temperatures, leaf internal CO_2_, and economic feasibility of cutting production, are presented in the **Supplementary Material File**.

**Table 3:** Mean ± standard deviation for whole canopy and ratios of *Cannabis sativa* ‘Suver Haze’ at four cumulative solar and LED lighting levels in 21 days (∑PPFD).

∑PPFD (mol m^-2^)	Shoot fresh mass (g m^-2^)	Shoot dry mass (g m^-2^)	Water per dry mass (g g^-1^)	Stems (%)	Leaves (%)	
376 ± 49	602 ± 73	102 ± 14	4.9 ± 0.1	37 ± 2	63 ± 2	
625 ± 08	956 ± 87	161 ± 19	5.0 ± 0.2	38 ± 2	62 ± 2	
829 ± 59	1281 ± 114	223 ± 20	4.8 ± 0.0	39 ± 1	61 ± 1	
1088 ± 55	1610 ± 273	284 ± 56	4.7 ± 0.1	40 ± 1	60 ± 1	
	L	L	L	L	L	
∑PPFD (mol m^-2^)	Main stem height (cm)	N° of nodes on the main stem	Internode length (cm)	Main stem diameter (mm)	LAI (m^2^ m^-2^)	SLW (g m^-2^)
376 ± 49	52.0 ± 3.8	17 ± 1	3.11 ± 0.06	7.4 ± 0.2	2.1 ± 0.2	31.3 ± 0.7
625 ± 08	55.3 ± 0.9	18 ± 0	3.08 ± 0.10	8.9 ± 0.1	2.8 ± 0.3	35.1 ± 1.1
829 ± 59	57.9 ± 2.9	19 ± 1	3.08 ± 0.20	10.3 ± 0.5	3.6 ± 0.3	37.7 ± 1.2
1088 ± 55	55.9 ± 1.4	20 ± 1	2.85 ± 0.14	11.1 ± 0.9	4.4 ± 0.7	38.6 ± 0.7
	NS	L	L	L	L	Q
∑PPFD (mol m^-2^)	Total branches m^-2^	1° branches m^-2^	2° branches m^-2^	Chlorophyll index	Flavonol index	Anthocyanin index
376 ± 49	218 ± 70	136 ± 9	81 ± 61	32.2 ± 1.1	1.11 ± 0.03	0.122 ± 0.004
625 ± 08	287 ± 29	142 ± 9	145 ± 26	33.7 ± 1.0	1.26 ± 0.02	0.128 ± 0.002
829 ± 59	390 ± 34	160 ± 2	230 ± 32	36.1 ± 1.2	1.39 ± 0.03	0.138 ± 0.002
1088 ± 55	508 ± 37	164 ± 11	344 ± 34	35.9 ± 1.0	1.43 ± 0.07	0.144 ± 0.006
	L	L	L	L	Q	L
∑PPFD (mol m^-2^)	Water use (L m^-2^)	WUE_Shoot_ (g L^-1^)	WUE_Total branches_ (Branch L^-1^)	WUE_1° branches_ (Branch L^-1^)	WUE_2° branches_ (Branch L^-1^)	
376 ± 49	39.8 ± 3.4	2.6 ± 0.3	5.5 ± 1.7	3.4 ± 0.3	2.2 ± 1.2	
625 ± 08	51.1 ± 9.5	3.2 ± 0.5	5.8 ± 1.4	2.9 ± 0.7	2.9 ± 0.8	
829 ± 59	58.7 ± 3.8	3.8 ± 0.5	6.7 ± 0.9	2.7 ± 0.2	3.9 ± 0.7	
1088 ± 55	70.6 ± 10.3	4.1 ± 0.9	7.3 ± 1.4	2.4 ± 0.5	5.0 ± 0.9	
	L	L	NS (p=0.06)	L	L	

Results in square meter (m^2^) can be expressed in plant basis by dividing them by density (10.8 plant m^-2^). Shoot = leaf and stem biomass. LAI, leaf area index; SLW, specific leaf weight; 1° and 2°: primary and secondary; WUE, water use efficiency. % are on shoot biomass basis.

L and Q (p<0.05) for linear (L) and quadratic (Q) terms in linear regression analysis; NS (p≥0.05). In all cases, N=12, n=3, and the number of observational units varied, as described in Material and Methods.

### Crop growth, development, and branch production

Plant and crop growth, development, and morphological parameters were evaluated under different greenhouse lighting conditions (**Table 1**) to characterize the overall plant quality, aiming to produce plants for flower induction and plants for the production of cuttings (stock plants).

**Figure 1:**
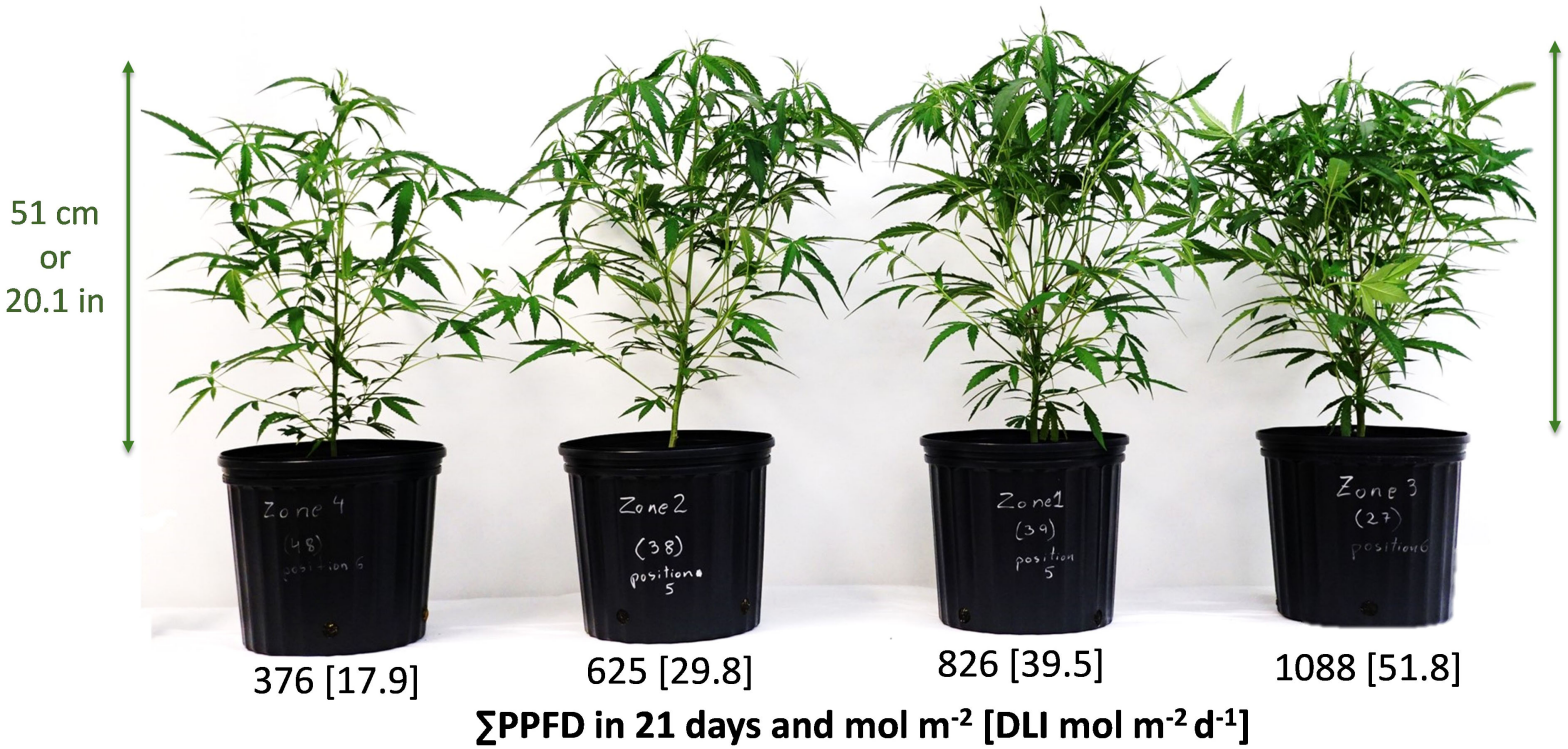
Plot or zone representative *Cannabis sativa* ‘Suver Haze’ plants grown in a greenhouse after 20 days of four supplemental LED light levels (151, 300, 501, and 703 µmol m^-2^ s^-1^). Numbers represent the cumulative PPFD and [average DLI] from the LED treatment and sunlight in 21 days at the top of the canopy.

**Figure 2:**
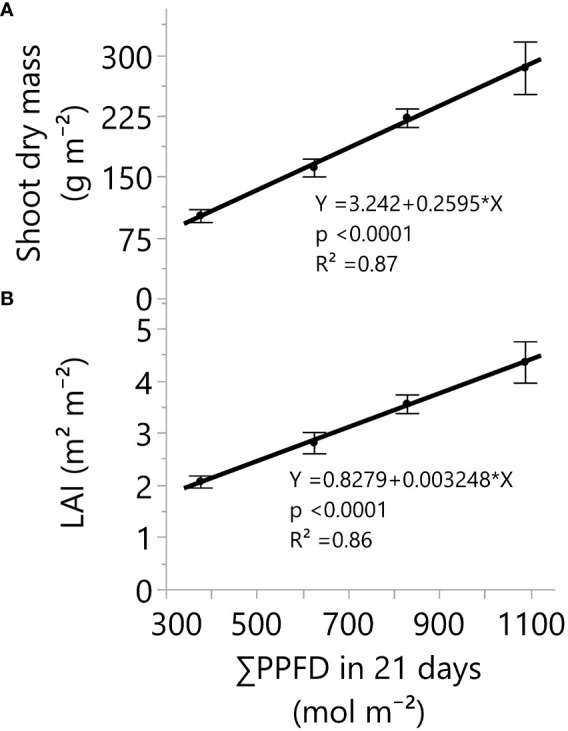
Shoot dry mass **(A)** and leaf area index **(B)** responses under four greenhouse cumulative light levels (sunlight plus supplemental LED light). PPFD represents photosynthetic photon flux density. p-values of the slopes (0.26 g m^-2^ and 0.003 m^2^ m^-2^) from linear regression analyses. Dots and error bars represent the mean and standard errors for each light treatment group, respectively. In all cases, N=12, n=3, and OU=3 plants per plot.

**Figure 3:**
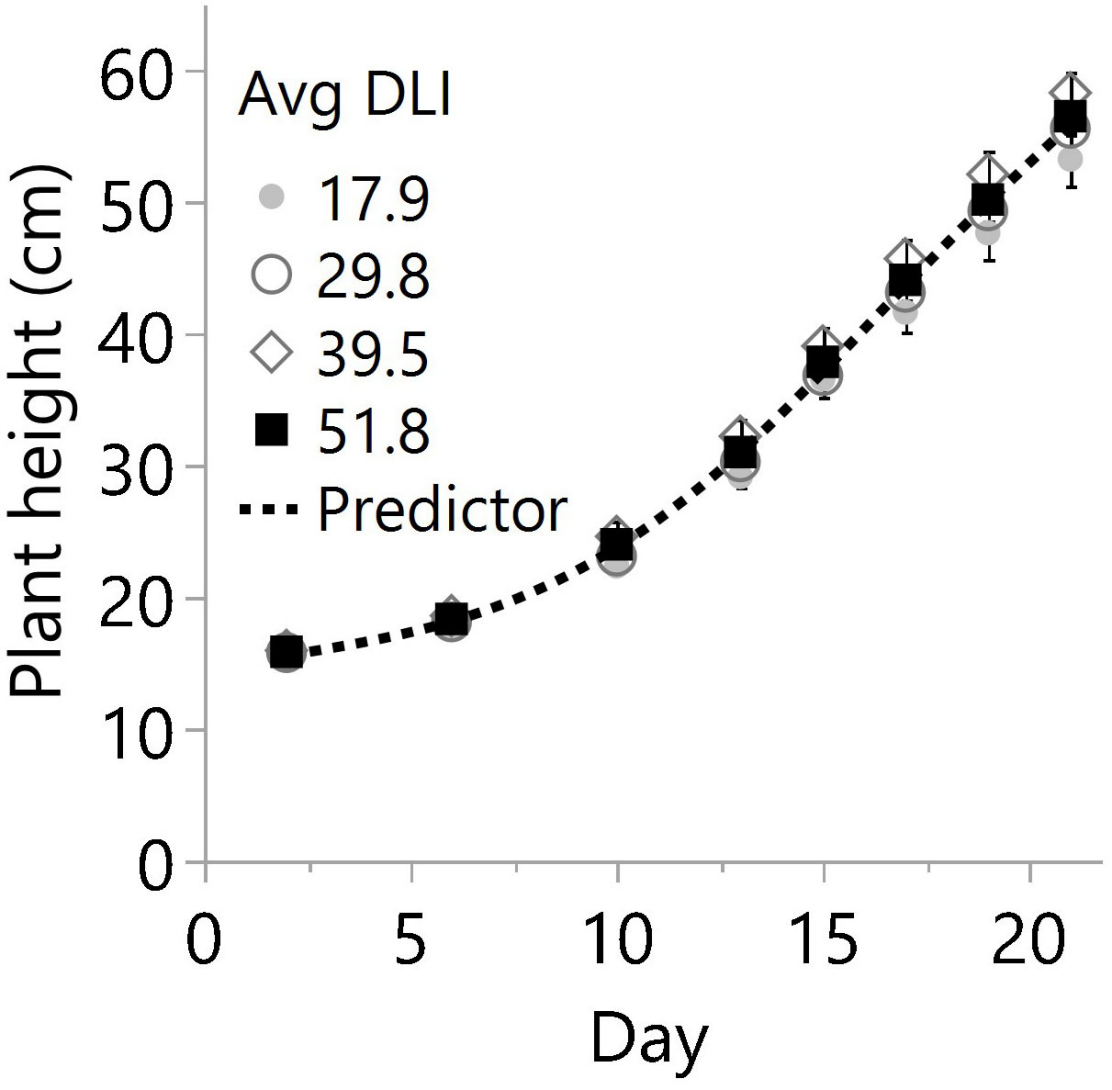
Plant heights on different days after planting in the greenhouse for four average daily light integrals (DLI) expressed in mol m^-2^ d^-1^. Every mark represents the average of 18 plants per date, and bars are the standard error from 3 plots (N=12, n=3, OU=6 plants per plot and date). For each date, linear regression and ANOVA indicated no differences in height from additional lighting (p≥0.075) or between specific light levels (p≥0.139), respectively. Therefore, the overall predictor model based only on days after planting is 14.08+((69.20-14.08)/(1+e^-0.25*(Day-69.20)^)) with R^2^ 0.983.

**Figure 4:**
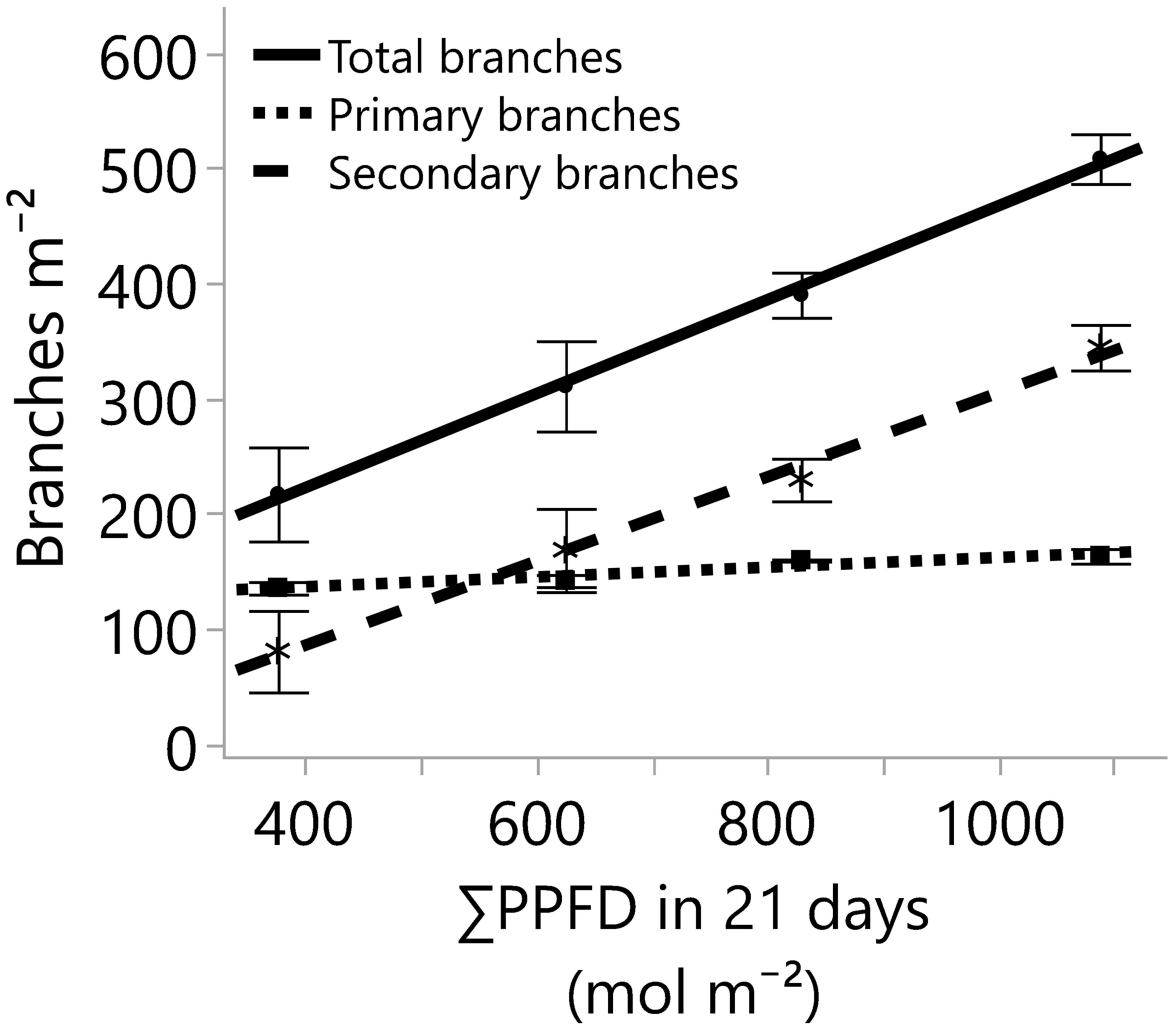
The number of branches developed on the main stem (primary branches) and on the primary branches (secondary branches), and the total number of branches (primary and secondary branches) for four greenhouse cumulative light levels (sunlight plus supplemental LED lighting). PPFD represents photosynthetic photon flux density, and the lines represent the regression equations for the primary (Y=119.7 + 0.04235*X; R^2^ 0.67), secondary (Y=-59.94 + 0.3647*X; R^2^ 0.84), and total branches per square meter (Y=59.72 + 0.407*X; R^2^ 0.85). In all cases, the p-values of the linear regression slopes were statistically significant (≤0.001). Dots, stars, and squares represent the means, and error bars represent standard errors for each light treatment group. In all cases, N=12, n=3, and OU=3 plants per plot.

Greenhouse lighting from ~18 to 52 mol m^-2^ d^-1^ led to linear increments in fresh mass, dry mass, number of nodes, internode length reduction, stem diameter, leaf area index (LAI), and branches (**Table 3**, **Figures 1**–**4**). In addition, increases in light intensity led to quadratic increments in specific leaf weight (SLW). Notably, under solar and supplemental light intensities between ~700 and 2000 µmol m^-2^ s^-1^ (**Supplementary Figure S3B**), along with an LAI expansion from 0.04 (initial leaf area) to 4.4 m^2^ m^-2^ (**Table 3**), the vegetative growth of ‘Suver Haze’ did not exhibit a growth saturation or light efficiency reduction (g mol^-1^) even when exposed to an average DLI of 52 mol m^-2^ d^-1^. More specifically, the crop’s dry mass linearly increased at a rate of 0.26 g mol^-1^ from 102 to 376 g m^-2^ (**Figure 2A**). In a similar way, the LAI linearly increased from 2.1 to 4.4 m^2^ m^-2^ (**Figure 2B**; **Table 3**). Furthermore, higher DLIs and LAIs were generally accompanied by increased SLW (specific leaf weight) and top-leaf metabolite concentrations per area (**Table 3**).

Despite differences in light quantity and quality (**Tables 1**, **2**), the internode length and plant height exhibited marginal or non-significant responses to the light treatments (**Table 3**; **Figure 3**). On the other hand, the numbers of primary, secondary, and total branches linearly increased with more light. However, branching rates displayed a significant contrast; primary branches registered a rate of 0.04 branch mol^-1^ m^-2^, while secondary branches exhibited a substantially higher rate of 0.36 branch mol^-1^ m^-2^ (**Figure 4**) with the increase in light intensity. This difference highlighted the branching dynamics in response to light, with secondary branches surpassing primary branches by nine times.

In conclusion, all the results and their statistical analyses support our hypothesis that increments in the cumulative light dosage would elicit an increase in plant growth and the number of branches. In addition, there was no evidence of light saturation.

### Crop water use and efficiency

**Figure 5:**
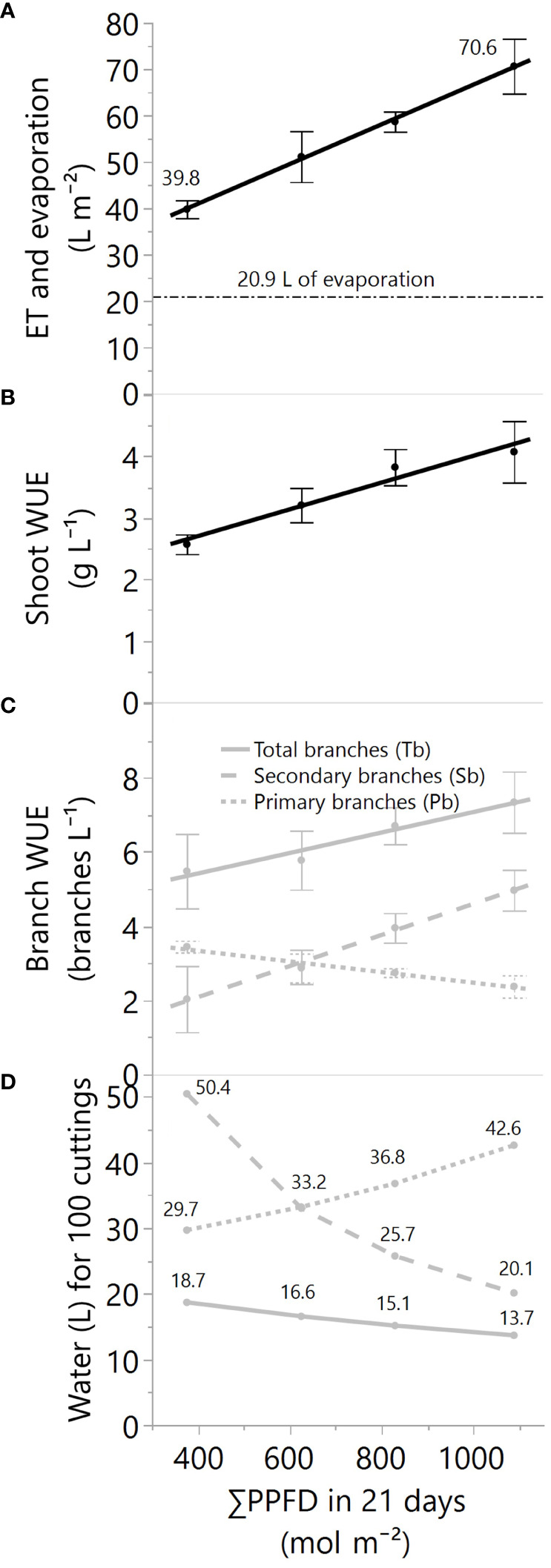
**(A)** The total evapotranspiration or ET (black solid line) and evaporation (black dashed line) in liters per square meter, **(B)** the plant-crop water use efficiency in grams of dry mass per liter of evapotranspired water, **(C)** the WUE for primary, secondary, and all branches, and **(D)** the water needs for 100 cuttings while increasing the greenhouse supplemental light levels. All values are for 21 days of greenhouse production, and PPFD represents photosynthetic photon flux density. For data in panels **(A–C)**, the linear regression fitting equations, R^2^, and p-values of the equation slopes are **(A)** ET=23.79 + 0.0429*X, 0.77, p<.01, **(B)** plant WUE=1.83 + 0.0022*X, 0.58, p<.01, and **(C)** Tb-WUE=4.31 + 0.0028*X, 0.29, p=.06, Pb-WUE=3.91 -0.0014*X, 0.49, p=.01, Sb-WUE=0.41 + 0.0042*X, 0.62, p<.01. Dots and error bars represent the mean and standard errors for each light treatment group, respectively. In all cases, N=12, n=3, and OU=1-2 load-cell readings per plot for evapotranspiration and 3 plants per plot for biomass and branches.

The water usage (evapotranspiration in 21 days) and water use efficiencies (WUE) to produce shoots and branches were quantified from ~18 to 52 mol m^-2^ d^-1^ (DLI). Light supplementation linearly increased the cumulative crop evapotranspiration from 39.8 to 70.6 liters (L) per square meter (m^-2^) (**Figure 5A**). Simultaneously, the pot evaporation was measured at 20.9 L m^-2^, representing transpiration rates between ~48 to 70% of the evapotranspiration. Despite higher water losses under more light, the shoot WUE increased linearly from 2.7 to 4.2 g L^-1^ by increasing the supplemental lighting (**Figure 5B**).

At the branch level, the WUE varied based on the type of branches (**Figure 5C**) and the branch response to the light (**Figure 4**). For instance, the WUE of primary branches was reduced with light augmentation (**Figure 5C**) because of the lower degree of light-level dependency of primary branches (**Figure 4**). However, the increase of light substantially enhanced the WUE of secondary branch production, elevating it from 2 to 5 branches per liter (**Figure 5C**). This improvement can be attributed to the more pronounced impact of light on secondary branches compared to its influence on primary branches. Consequently, the combined results of WUE for total branches, primary plus secondary branches, suggest a reduced but still considerable effect by the light augmentation, with WUEs from 5.5 to 7.3 for total branches per liter of water (p=0.06, **Figure 5C**; **Table 3**). Nevertheless, the water needs for cutting production can exhibit significant variability, ranging from approximately 14 to 19 liters of water for every 100 cuttings for highly efficient growers who utilize all available branches to approximately 20 to 50 liters of water for every 100 cuttings for those who selectively employ smaller or larger caliper cuttings (**Figure 5D**).

### Leaf photosynthesis, pigments, stomata, transpiration, and WUE

Top leaves of plants grown under ~18 and 52 mol m^-2^ d^-1^ (DLI) were subjected to a range of short-term light intensities from 150 to 2000 µmol m^-2^ s^-1^ (PPFD) to analyze the impact of long-term greenhouse lighting (DLI) on net photosynthesis, transpiration, stomatal conductance, and leaf WUE. Additionally, chlorophyll, flavonol, and anthocyanin indexes (light pigments) were quantified.

**Figure 6:**
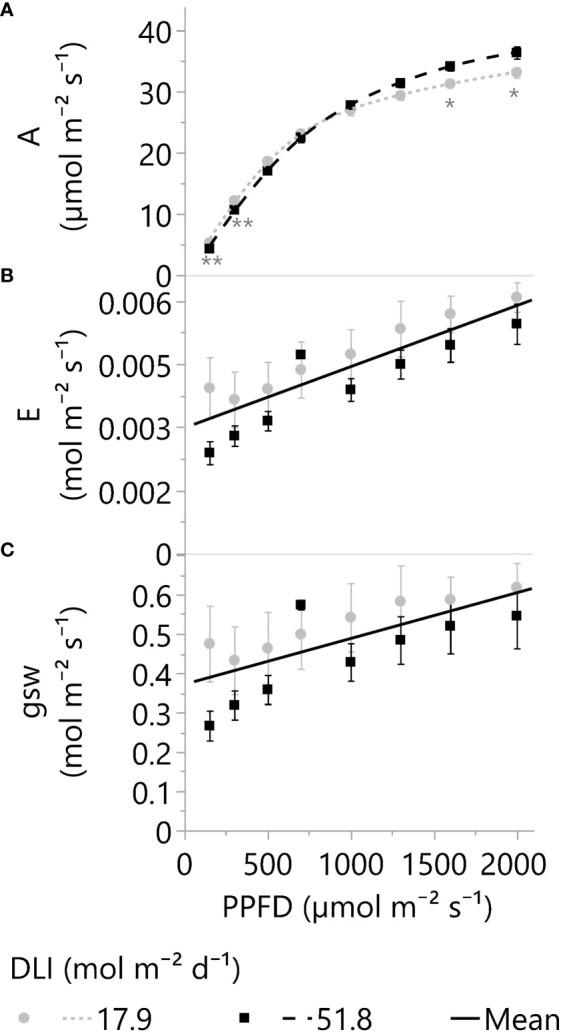
**(A)** Net photosynthetic rates or A, **(B)** transpiration rate or E, and **(C)** stomatal conductance or g_sw_ of leaves adapted to greenhouse DLI levels of ~18 or 52 mol m^-2^ d^-1^. Young, fully expanded, and light-exposed leaves from both DLI treatments were evaluated at PPFDs of 150, 300, 500, 700, 1000, 1300, 1600, and 2000 µmol m⁻² s⁻¹ (Blue 18% and Red 82%) using a Li-6800 + 6800-01A. Asterisks indicate differences between the two DLI treatments at each PPFD level by one (*) or two (**) tailed T-tests (α 0.05). Split-plot analysis for E and g_sw_ (factors: DLI, PPFD, Time of the day, and DLI*PPFD) indicates differences only because of the PPFD (p <0.001). The E and g_sw_ linear equations (solid lines) are 2.984e^-3 + ^1.45e^-6^*PPFD and 0.3715 + 1.16e^-4^*PPFD, respectively. Dots and squares represent the mean, and error bars represent the standard errors for each DLI-PPFD combination. In all cases, N=6, n=3, and OU=1 leaf per plot.

The leaf photosynthetic rates (A) of plants grown under 18- and 52-DLI treatments increased even under 2000 µmol m^-2^ s^-1^, but the increments in A with more PPFD were dimmed and only 6.2 ± 1.9% (mean ± SD) higher in 2000 than in 1600 µmol m^-2^ s^-1^ for both DLI treatments (p<0.001). As a reference, the average proportional increments in A (and PPFD) based on 2000 µmol m^-2^ s^-1^ were 14% (150), 30% (300), 51% (500), 66% (700), 79% (1000), 87% (1300), 94% (1600), and 100% (2000). Furthermore, small photosynthetic differences between DLI treatments were observed under low and high PPFDs; for instance, 52-DLI leaves exhibited higher photosynthetic rates when exposed to short-term PPFDs exceeding 1300 µmol m^-2^ s^-1^, but photosynthesis was higher under 18-DLI leaves when PPFDs dropped below 500 µmol m^-2^ s^-1^ (**Figure 6A**). Additionally, leaves grown under 52 mol m^-2^ d^-1^ presented higher concentrations of photosynthetic (chlorophylls) and non-photosynthetic pigments (flavonols and anthocyanins) compared to 18 mol m^-2^ d^-1^ (**Table 3**).

Unlike photosynthesis, leaf transpiration (E) and stomatal conductance (g_sw_) increased linearly with PPFD (**Figures 6B, C**). However, increments in stomatal and transpiration rates were not proportional to the light levels. For example, the transpiration rate per photon of light under 2000 µmol m^-2^ s^-1^ was 7.3 times lower than at 150 µmol m^-2^ s^-1^ or 2.9 vs 21.3 µmol H_2_O µmol photon^-1^, respectively. Furthermore, despite the raised g_sw_ and E with short-term increments in PPFD, there were no statistical differences by long-term light levels, 18 vs. 52 mol m^-2^ d^-1^ (p ≥.29; **Figures 6B, C**).

In concordance with the crop WUE, the leaf WUE of upper leaves, or A/E, increased up to a PPFD of 700 µmol m^-2^ s^-1^ (p<0.001); differences in WUE were not statistically significant from 700 to 2000 µmol m^-2^ s^-1^ (α=0.05). Moreover, the main DLI effect (~18 vs. 52 mol m^-2^ s^-1^) on leaf WUE or the interaction DLI*PPFD was not statistically significant (α=0.05). Therefore, there was no evidence to support this study’s second hypothesis: Leaves of cannabis plants cultivated under an elevated DLI would display higher WUE due to increased photosynthetic rates than their counterparts exposed to a lower DLI when leaves were evaluated under high PPFDs.

## Discussion

### Crop growth and development under greenhouse lighting and its effects on water use efficiency

#### Light effects and DLI recommendations on plant growth and production

The effects of light on plant growth can exhibit substantial variation across plant species, cultivars, developmental stages, and environmental factors, including plant nutrition, water availability, light intensity, and spectra. This variance stems from the diverse photosynthetic capacities inherent to different species, as well as the intricate interplay of multiple environmental factors (Boyer, 1970; Penning de Vries et al., 1989; Rosati and Dejong, 2003; Zhao et al., 2003; Weaver and Van Iersel, 2020). For instance, cultivating crops under identical DLI values but with lower light levels and extended photoperiods can lead to equivalent or even heightened daily growth. This is attributed to the enhanced efficiency of photosynthesis at both the leaf level (Weaver and Van Iersel, 2020) and the crop level (Hui et al., 2001). Furthermore, light wavelengths outside the Photosynthetically Active Radiation (PAR) range, 400 – 700 nm, can significantly influence growth and development. Far-red light (700 to 800 nm), for instance, promotes leaf expansion and photosynthesis in the 700-750 nm range (Zhen et al., 2021). In light of these findings, an extended PAR range (ePAR) from 400 to 750 nm has been proposed to account for photosynthesis enhancements (Zhen et al., 2021). Therefore, due to the complex interplay of these factors, comparing and adopting light levels across different situations is challenging and should ideally be confined to the specific conditions under evaluation. Nevertheless, for general reference, we provide a comparison with other cannabis studies, crops, and indoor systems employing the conventional DLI range (DLI 400-700nm) while offering the extended range values as a reference (**Table 1**).

Within the realm of cannabis cultivation, this study, as well as prior investigations employing exclusive LED lighting sources (Moher et al., 2021; Rodriguez-Morrison et al., 2021), provides compelling evidence to classify cannabis among the most elevated lighting requiring crops. For comparison, optimal lettuce production is associated with a maximum of 17 mol m^-2^ d^-1^ for marketable leaf growth, with higher light levels potentially leading to increased biomass but also an elevated risk of leaf tip burn (Both, 2002). High-light greenhouse crops like tomatoes and cucumbers require at least 30 mol m^-2^ d^-1^ for optimal yields and fruit quality (Dorais et al., 2017), although significant research into higher light levels may not have been pursued due to economic feasibility constraints for production (van Iersel and Gianino, 2017). In corn breeding, supplemental greenhouse lighting is recommended to reach up to 750 µmol m^-2^ s^-1^ or ≥38 mol m^-2^ d^-1^, depending on the photoperiod, while corn research in chamber conditions employs as much as 1000 µmol m^-2^ s^-1^ or 58 mol m^-2^ d^-1^ (Eddy and Hahn, 2010). Remarkably, indoor farming studies have shown that cannabis crops can utilize 82 mol m^-2^ d^-1^ for shoot growth (Moher et al., 2021) and 78 mol m^-2^ d^-1^ for flower production (Rodriguez-Morrison et al., 2021).

Despite growth increments in the vegetative stage of cannabis under high light, the light-to-biomass conversions in Moher et al. (2021) were lower than in our study. For instance, the biomass yield in the Moher et al. (2021) study was 246 g m^-2^ with 52 mol m^-2^ d^-1^, and it increased to 271 g m^-2^ with 82 mol m^-2^ d^-1^. In contrast, our study yielded an average of 285 g m^-2^ with 52 mol m^-2^ d^-1^ (eDLI: 53; **Table 1**) and showed no indications of light saturation (**Figure 2A**); importantly, both studies shared a consistent 21-day timeframe, facilitating a valid DLI-based comparison. It is plausible that the improved greenhouse study result in our research could be attributed to differences in plant genetics (Vanhove et al., 2012; Suchoff et al., 2021), light spectra (Lalge et al., 2017; Zhen et al., 2021), temperature conditions (Chandra et al., 2008, 2011), and/or the effects of water and fertilizer supply. Nevertheless, it is evident that further research into cannabis cultivation and production conditions is necessary to gain a deeper understanding and achieve consistent maximum yields and product quality.

#### Light effects on LAI

In this investigation, plants were uniformly spaced at intervals of 30.5 cm, resulting in a plant density of 10.8 plants per square meter. In crop production, manipulating plant densities is a strategy to optimize yields and cost-efficiency by capitalizing on resource utilization and enhancing the efficacy of lighting, cooling, and heating per unit area. For instance, elevating plant density leads to an early expanded leaf canopy and improved light interception capacity. Moreover, an optimal leaf area is key to increasing crop photosynthesis and water use efficiency (Hui et al., 2001). However, an excessive plant density does not necessarily translate to increased yields, as seen in cannabis cultivation (Vanhove et al., 2012). The Leaf Area Index (LAI) assesses light interception and quantifies the total leaf area relative to the ground area (m^2^ m^-2^). Previous research across multiple plant species has consistently reported that an LAI in the range of 3 to 4 intercepts approximately 95% of the incident light, whereas an LAI of 2 captures roughly 80% (Shibles and Weber, 1965; De Visser et al., 2007; Brodrick et al., 2013; Tabarzad et al., 2016). Given that an LAI of 3 to 4 represents an optimal threshold for light capture, and values exceeding four may not confer any significant advantages in terms of light interception (De Visser et al., 2007; Brodrick et al., 2013; Tabarzad et al., 2016) and growth (Shibles and Weber, 1965), growers are likely to enhance production efficiency by adjusting plant spacing when the LAI surpasses four. Consequently, for a duration exceeding 21 days and high-light conditions like those presented in this study (leading to an LAI of 4.4), plant density could be reduced or branches trimmed while still maintaining a critical light interception of around 95%. Conversely, for crops subjected to lower light levels (**Figures 1**, **2B**), pruning for harvesting cuttings or conducting formative pruning for flower production may have a negative impact on the LAI and plant growth.

#### Light effects on plant height and internode elongation

Responses of plant height in conjunction with internode extension are key to understanding whether the plant stretches mainly because of growth or to compete for more light, triggering shade avoidance syndrome or SAS (Kump, 2020).

In this study, the combination of light quantity and quality (**Tables 1**, **2**), did not lead to significant changes in plant height across various light treatments (**Figures 1**, **3**). Still, the light level and spectrum affect SAS responses (Kump, 2020), and perhaps they limited the observed differences in plant height in this study. For example, the lower light condition treatments may have contributed to some stem and internode extensions and vice versa. Furthermore, lower red to far-red ratios have increased stem length in several plant species (Demotes-Mainard et al., 2016). For example, in cucumber and lettuce, stem elongation was only significant when far-red light exceeded 9% at a DLI of 11.5 mol m^-2^ d^-1^, while the effects were marginal under 29 mol m^-2^ d^-1^ (Kusuma and Bugbee, 2023). In the present cannabis study, the daily average far-red ranged from 8% to 3% across DLIs of 18 to 52 mol m^-2^ d^-1^. Therefore, significant light spectrum effects would not be expected.

The overall findings and data suggest that the specific light conditions implemented (as outlined in **Tables 1** and **2**) had only a minor influence on plant height and internode length. These characteristics would be more substantially influenced by plant genetics and developmental factors than light intensity for cannabis.

#### Light effects on branching and development

Cannabis branches are fundamental in enhancing cutting production and supporting flowers. To assess the impact of light quantity on branch development, the total number of branches was measured and categorized into two groups: primary branches, which develop directly on the main stem, and secondary branches, which sprout from primary branches. The formation, dormancy, and outgrowth of buds that will give place to new stems, leaves, flowers, and branches are regulated by external and internal factors such as genetics, apical dominance, photoperiod, temperature, nutrition, and the amount and quality of the light, among others (Lortie and Aarssen, 1997; Cooke et al., 2012; Leduc et al., 2014). Among those factors, increasing the quantity of light plays a crucial role in reducing SAS responses like internode extension and axillary bud dormancy, while promoting bud outgrowth and overall growth in various species (Gommers et al., 2013; Leduc et al., 2014). Furthermore, the striking similarities in the main stem heights (**Figure 3**) and differences in primary and secondary branches (**Figure 4**) suggest that photochemical resources were strongly prioritized and influenced by the main apical meristem. The impact of this effect can be so pronounced that, in certain species or under specific growth conditions, the strong control of branching by the shoot apex may only cease upon removal (Lortie and Aarssen, 1997). Notably, in our study, cannabis branching proliferation was effectively achieved from ~18 to 52 mol m^-2^ d^-1^ (**Figure 4**) while maintaining a water and nutrient supply upon plant demand.

#### Light effects on crop water use and efficiency

The effects of increased supplemental lighting on water use and water use efficiency (WUE) were investigated to understand how water is used at a whole plant and production unit (cutting) in a 21-day production cycle. The primary increases in crop WUE observed in the present study are likely attributed to the pronounced benefits of enhanced photosynthesis at both leaf and crop levels, outweighing the potential drawbacks of increased transpiration under increasing lighting conditions. The increment in WUE at the crop level correlated with higher WUE at the leaf level, which is discussed later in this study. The findings of this study are corroborated by prior research conducted on greenhouse-grown sunflowers, wherein continuous measurements of crop photosynthesis and water use were undertaken under greenhouse solar conditions. In this previous study, it was observed that crop WUE throughout the day aligned closely with solar radiation levels, with peak values coinciding with the highest levels of solar radiation and photosynthesis (Hui et al., 2001).

Despite the correlations observed between branches and WUE (**Figure 5C**), it is imperative to note that the improvement in water efficiency is likely primarily attributable to impacts on photosynthesis and transpiration rather than the specific type of branches.

#### Crop water use and efficiency in cutting and flower production

Currently, there exists no standardized protocol for growers and researchers to systematically select the most optimal branch for propagating cuttings. Instead, cutting selection predominantly relies on individual preferences. In order to address the various potential scenarios, **Figure 5D** elucidates the relationship between branch selection and water usage under different light conditions. Based on these findings, on average, a nursery crop operating under conditions similar to those in this study is anticipated to require between 20 and 33 liters of water for every 100 cuttings, assuming that primary branches are mainly used to produce cuttings from secondary branches. Moreover, it’s noteworthy that the water demand per cutting is projected to be higher under lower greenhouse lighting levels (<18 mol m^-2^ d^-1^).

For flower producers, the shoot WUE results imply that to grow the same shoot biomass in 21 days, growers need 0.37 L g^-1^ under 18 mol m^-2^ d^-1^ of light and only 0.24 L g^-1^ under 52 mol m^-2^ d^-1^, a 35% water reduction in evapotranspiration. Similar effects were found in a greenhouse hydroponic lettuce crop that transpired about 0.5 L g^-1^ under 8 mol m^-2^ d^-1^ and 0.27 L g^-1^ under 22 mol m^-2^ d^-1^, a 46% water reduction in transpiration (Both, 2002).

### Influence of greenhouse supplemental lighting on photosynthesis, stomatal conductance, transpiration in leaves, and their interconnection with Water Use Efficiency

Anatomical and physiological leaf characteristics change based on the prevailing light environment (Chabot and Chabot, 1977; Feng et al., 2019). Leaf photosynthesis (A), stomatal conductance to water vapor (g_sw_), and transpiration (E) are integral to understanding growth and water usage and efficiency at the leaf level while helping elucidate the broader responses at the crop level.

#### Photosynthetic responses and significance

This study’s results align with similar findings reported for cannabis cultivation under sole-source LED lighting, which exhibited stronger photosynthetic responses and DLI variations (Rodriguez-Morrison et al., 2021). Furthermore, higher leaf maximum-A capacities, as seen in this study and Rodriguez-Morrison et al. (2021), are expected under light augmentation due to increments in leaf weight per area (Penning de Vries et al., 1989). Thicker or denser leaves can produce more RuBisCO (Penning de Vries et al., 1989) and chlorophyll in the same area (**Table 3**), potentially increasing photosynthesis (Emerson, 1930). On the other hand, higher respiration rates, driven by the elevated biomass and metabolite levels per area (**Table 3**), may provide an explanation for the lower net photosynthetic rate observed in thicker leaves in cannabis research. In summary, our leaf photosynthetic rates results support the remarkable crop yield increments even at maximum greenhouse radiances of ~2000 µmol m^-2^ s^-1^ (**Supplementary Figure S3B**).

#### Stomatal and transpiration responses and regulation

In this study, the observed rise in E with increased light intensity was attributed to the increased stomatal conductance (g_sw_). Typically, a higher g_sw_ is linked to either a reduction in leaf internal CO_2_ concentration (Ci) or an enhancement in photosynthesis (A) through light supplementation (McAusland et al., 2016; Driesen et al., 2020). However, despite the linear increase in g_sw_ with light intensity, the responses of A and Ci did not parallel the stomatal conductance trend observed in our study (**Figures 6C, A**; **Supplementary S4A**).

The linear response in g_sw_ compared to the plateau response of A could be explained by additional mechanisms affected by light other than C_i_ reduction or photosynthesis enhancement. For instance, blue and red radiation is mentioned as direct influencers on guard-cell water status (Driesen et al., 2020), while shortwave radiation, encompassing most or all solar wavelengths, demonstrated an impact on leaf water status (Lawson et al., 2010; Pieruschka et al., 2010). Pieruschka et al. (2010) proposed a regulatory mechanism where the balance between radiation-driven water vapor production and transpiration rate governs stomatal conductance. Moreover, their observations found no specific wavelength effects, establishing only a linear correlation between stomatal conductance and absorbed radiation energy. Another factor affected by radiation is leaf temperature, a pivotal influence in stomatal conductance. Feller (2007) and Urban et al. (2017) demonstrated significant increments in stomatal aperture across temperatures ranging from ~20°C to over 40°C. Moreover, these increments persisted despite reductions in A and water potential and an increase in C_i_ (Urban et al., 2017). In the present cannabis study, results showed marginal leaf temperature differences (26.1 ± 0.17 to 26.9 ± 0.35°C) when elevating the PPFD from 150 to 2000 µmol m^-2^ s^-1^ (**Supplementary Figure S4B**). In conclusion, light can affect photosynthesis and transpiration differently and may act on the stomatal aperture via various mechanisms.

#### High stomatal conductance and water loss at low radiation levels

Our study found considerably higher E and g_sw_ values per photon of light at lower PPFDs than at higher light intensities, as previously shown in the result section. Moreover, leaf transpiration at 150 µmol m^-2^ s^-1^ constituted a noteworthy 57% of the total water loss found under 2000 µmol m^-2^ s^-1^ (**Figure 6B**). This can be explained by reports across diverse species (Hui et al., 2001; de Dios et al., 2015; Dayer and Gambetta, 2021; Dayer et al., 2021) that suggest rapid and excessive increments in stomatal opening at low light or in the absence of light, which can increase water losses. Furthermore, data from different species, spanning ferns, gymnosperms, and angiosperms, also revealed that at very low light levels (50 µmol m^-2^ s^-1^), stomatal conductance constituted between ~16% to 64% of the aperture at 1000 µmol m^-2^ s^-1^ (Deans et al., 2019). These results also align with increments above typical nighttime transpiration rates of 5% to 15% (Caird et al., 2007). Moreover, the observed rapid and predawn stomatal aperture increments have been associated with specific wavelengths (Lawson et al., 2010) and circadian regulation (de Dios et al., 2015; Dayer et al., 2021), while stomatal conductance did not correlate with photosynthesis at low light (Deans et al., 2019).

#### The benefits of enhancing supplemental lighting on leaf transpiration

In addition to increasing photosynthesis, enhancing supplemental lighting strongly reduced the water use per photon of light. These findings align with the broader context of light effects on stomatal conductance observed across various species (Deans et al., 2019), as previously discussed. Additionally, similar trends in crop transpiration have been documented in beans and cotton subjected to maximum PPFDs of 500 and 1500 µmol m^-2^ s^-1^. Notably, nocturnal water losses constituted 23% and 12% of daytime requirements under lower and higher PPFDs, respectively (de Dios et al., 2015). These results suggest that optimizing lighting conditions can significantly mitigate the impact of both night-time and day-time water losses.

#### Stomatal slow response implications

Stomatal conductance and transpiration exhibited linear increases by increasing PPFD, as detailed earlier. However, the absence of statistical evidence for DLI effects between 18 and 52 mol m^-2^ d^-1^ was unexpected. It is plausible that the lack of statistical differences is linked to the substantial variability in transpiration and stomatal conductance due to the interaction of our procedure’s sampling speed and stomatal acclimation periods (Lawson and Blatt, 2014; McAusland et al., 2016). Nevertheless, the information and results reviewed emphasize the complex interplay between direct and indirect light effects on guard cells, transpiration, and physiological dynamics, contributing to notable increases in gas exchange within the context of our research. Additionally, it is noteworthy that cannabis leaf temperatures remained similar to or below an air temperature of 27°C. This observation suggests that transpiration rates were sufficient to maintain optimal metabolic functions without the risk of overheating (Feller, 2007; Nelson and Bugbee, 2015). Our investigation revealed no physiological constraints on stomatal conductance and transpiration, even under the more demanding conditions of ~2000 µmol m^-2^ s^-1^ of actual greenhouse LED lighting and solar radiation (unshown data). This and previous research stress the robustness of optimal cannabis growth under high-light environments.

#### The impacts of short and long-term lighting exposure on leaf water use efficiency

**Figure 7:**
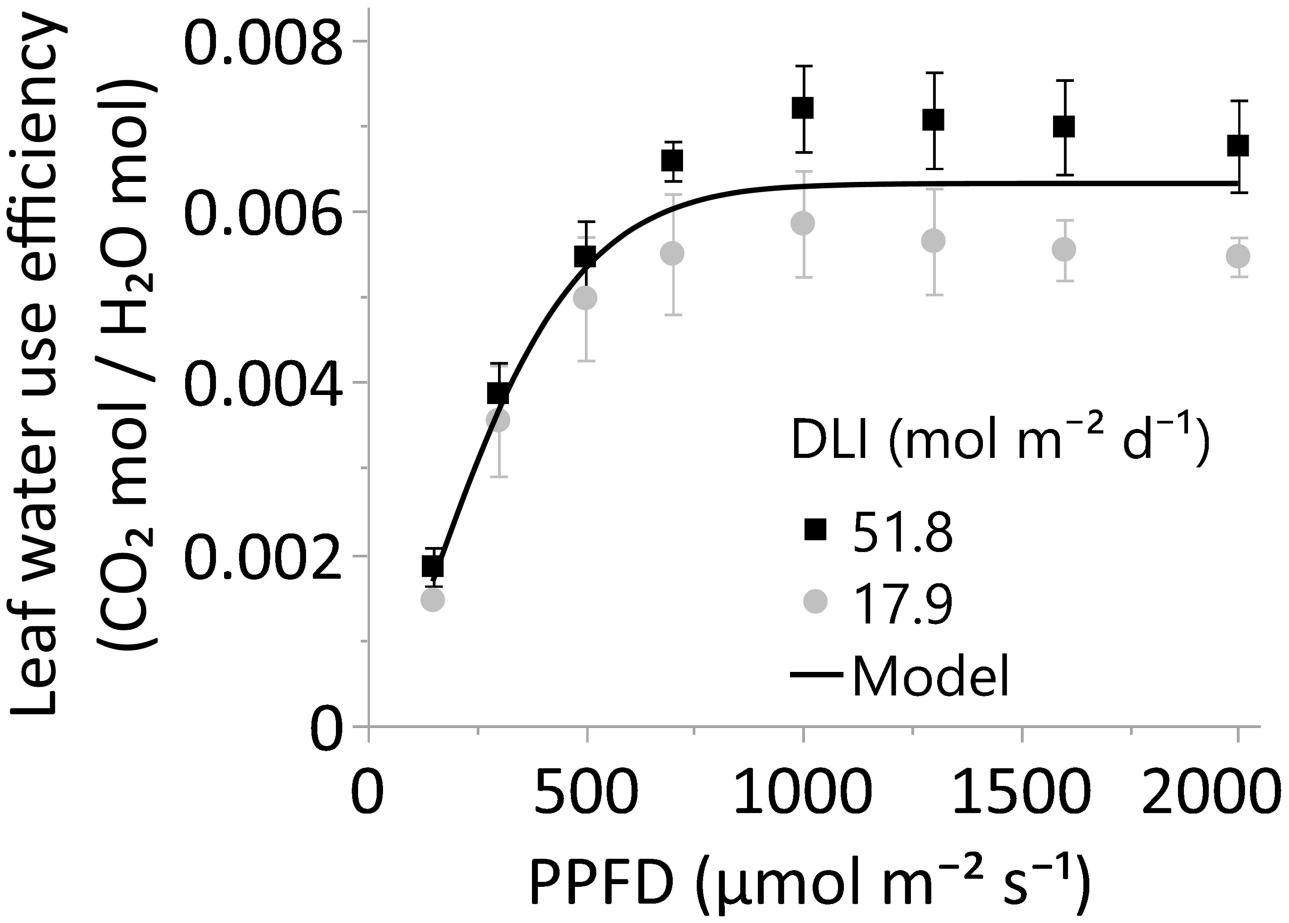
The leaf water use efficiency, or A/E, of leaves grown under greenhouse DLI levels of ~18 or 52 mol m^-2^ d^-1^. Young, fully expanded, and light-exposed leaves from both DLI treatments were evaluated at PPFDs of 150, 300, 500, 700, 1000, 1300, 1600, and 2000 µmol m⁻² s⁻¹ (Blue 18% and Red 82%) using a Li-6800 + 6800-01A. Comparison between DLI treatments at each PPFD level was performed by T-test. Differences between the two DLI levels were not statistically significant (α 0.05). The solid line represents the three-parameter model =0.0063*(1-e^-(PPFD/330.76)^1.49^). Equation decay parameters were not different from zero using the JMP Pro 17.0 four-parameter models. In all cases, N=6, n=3, and OU=1 leaf per plot.

The leaf WUE of upper cannabis leaves remarkably increased as a consequence of the curvilinear and linear responses observed for photosynthesis and transpiration (**Figures 6A, B**, and **7**). Consistent with our findings, Deans et al. (2019) reported a substantial increase in intrinsic WUE (A/g_sw_) under elevated lighting conditions across species, which was driven by a more pronounced increase in photosynthesis than stomatal conductance. In our study, there was weaker evidence indicating that the leaf WUE of the 52-DLI treatment might surpass that of the 18-DLI WUE under light levels of 1000 µmol m^-2^ s^-1^ or higher (p-values >0.05 to 0.09); however, at α 0.05, the statistical support was not robust enough to substantiate our hypothesis that leaves developed under the 52-DLI treatment exhibit higher WUE than leaves under the 18-DLI treatment.

In conclusion, the observed increase in crop WUE with higher lighting, consistent with other species, lighting conditions (Hui et al., 2001; Both, 2002; de Dios et al., 2015), and supported by increments in leaf WUE across various cannabis cultivars (unpublished data) and fifteen different species (Deans et al., 2019), suggests a potential for elevated WUE and yield in unstressed crops exposed to increased lighting conditions.

### Spectral differences between treatments

In the current study, variations in light intensity among treatments were achieved through incremental adjustments in blue and red LED lighting, while similar solar radiation was maintained across treatments (**Table 1**). Consequently, this led to an augmentation in the proportion of blue and red wavelengths relative to other spectrums within the greenhouse solar spectrum. It is noteworthy that differences in the spectral composition of electrical lighting can significantly impact plant physiology, morphology, and growth in growth chambers (Spalholz et al., 2020) and under conditions of low sunlight, 3-5 mol m^-2^ d^-1^ (Hernández and Kubota, 2014; Collado and Hernández, 2022).

While prior research has demonstrated substantial effects of supplemental light spectrum variations under very low sunlight conditions, the impact diminished under higher solar DLIs, ~12-16 mol m^-2^ d^-1^, in tomatoes and cucumbers (Hernández and Kubota, 2014; Kaiser et al., 2019). Furthermore, the influence of changing light quantity from 11.5 to 29 mol m^-2^ d^-1^ was more pronounced on the biomass of seven primary agricultural crops in growth chambers than alterations in light spectrum (blue, green, and red wavelength ranges: 10.8-27.5%, 1.7-48.0%, and 24.5-86.3%, respectively; a phytochrome photo-equilibrium (PPE) range: 0.83-0.89; Snowden et al., 2016). Similarly, the impact of increasing DLI from 8.7 to 19.7 mol m^-2^ d^-1^ outweighed the effects of modifying the blue and red light ratios in cucumbers (Hernández and Kubota, 2014). In our cannabis research, light exposure ranged from 17.9 to 51.8 mol m^-2^ d^-1^, while spectrum and PPE ranges (**Table 2**) were similar to those discussed.

In summary, this experiment does not provide evidence to suggest that spectral differences between treatments significantly influenced plant responses under greenhouse supplemental lighting conditions. Instead, the variations in plant responses can be primarily attributed to the differences in total Photosynthetic Photon Flux Density (∑PPFD). Overall, these findings imply that the effects of light spectrum variations may be relatively smaller, especially in the presence of a complete light spectrum, such as solar radiation.

## Conclusions

Enhancing supplemental lighting promoted increased photosynthesis and played a pivotal role in shaping the water use dynamics in cannabis leaves and crops. Most importantly, it could potentially enhance the WUE of most crops while promoting growth. Our findings emphasize the capacity of lighting management to optimize water use efficiency while presenting valuable implications for both research and practical applications in agriculture.

For instance, stakeholders in cannabis nurseries, flower production, and research settings can substantially improve plant growth, yield, and water use efficiency by incorporating supplemental lighting. Specifically, our study indicates a linear growth response within the range of ~18 to 52 mol m^-2^ d^-1^, suggesting that DLIs exceeding 52 mol m^-2^ d^-1^ could further enhance cuttings and plant growth and surpass existing literature expectations. It is crucial to highlight that these results and expectations are based on meticulous control of water, nutrients, and other growth factors and genetics. In this study, *Cannabis sativa* cv Suver Haze was employed and represents an average-performing cultivar (Suchoff et al., 2021), suggesting that genetic variability may increase or decrease the number of branches and overall plant growth per mole of light.

This study demonstrated notable increases in leaf and crop WUE with elevated light levels while enhancing CO_2_ assimilation. Historically, improving WUE without compromising production has been challenging, particularly considering the dependence of photosynthesis on stomata (Lawson and Blatt, 2014; Xing et al., 2022). Furthermore, studies on CO_2_ enrichment (Hui et al., 2001) and elevated atmospheric CO_2_ (Guerrieri et al., 2019) have also reported improvements in WUE; in alignment with the findings on light supplementation, the enhanced photosynthesis was found to be the main contributing factor. Ultimately, there is evidence showing that the combination of light and CO_2_ enrichment can lead to even greater enhancements in water use efficiencies (Hui et al., 2001).

## Data availability statement

The raw data supporting the conclusions of this article will be made available by the authors, without undue reservation.

## Author contributions

CC: Conceptualization, Data curation, Formal analysis, Investigation, Methodology, Resources, Validation, Visualization, Writing – original draft, Writing – review & editing. SH: Investigation, Methodology, Writing – review & editing. RH: Conceptualization, Data curation, Funding acquisition, Methodology, Project administration, Resources, Supervision, Validation, Visualization, Writing – review & editing.
