# Supplementary material for: Supplemental greenhouse lighting increased the water use efficiency, crop growth, and cutting production in Cannabis sativa
Source: Front Plant Sci. 2024 Jun 7;15:1371702. doi: 10.3389/fpls.2024.1371702 (PMC11190854; doi:10.3389/fpls.2024.1371702)
Supplement: Supplementary file 1 [file DataSheet_1.docx]

**Supplementary material**

**MATERIALS AND METHODS**

**Experimental design and greenhouse setup:**


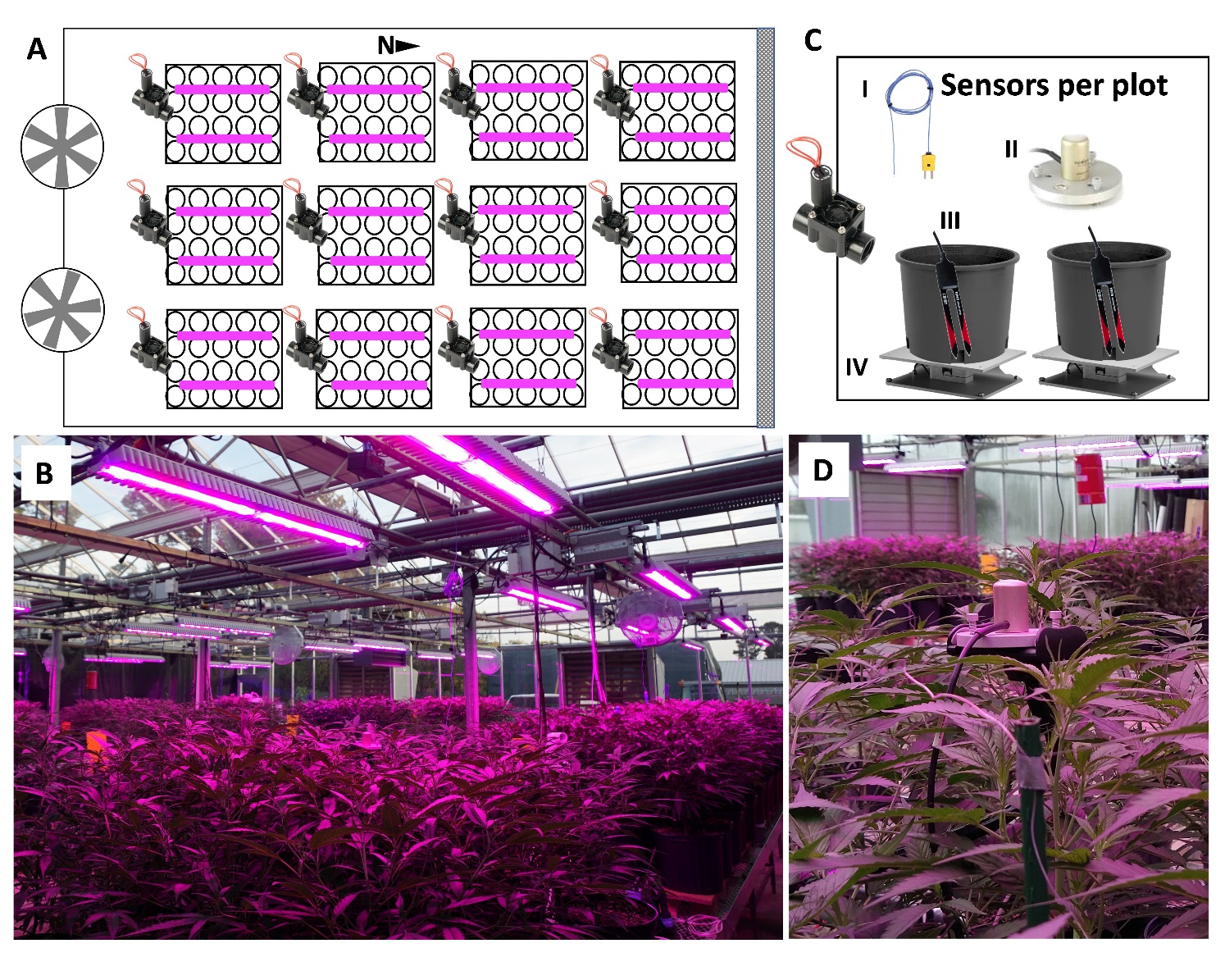
Figure S1. (**A**) Schematic representation showing the twelve plots (rectangles) with two LED fixtures per plot (purple lines), one solenoid valve, and 20 pots per plot (circles). Each light treatment was aleatorily assigned to three plots (n=3). Plot# from low to high light levels and their PPFD±SD (µmol of light m^-2^ s^-1^) were as follow: #4, 151±6; #7, 151±5; #9, 151±6; #2, 298±8; #8, 301±11; #12, 300±13; #1: 499±14; #6: 502±12; #11, 503±13; #3, 701±18; #5, 702±18; #10, 706±17. (**B**) A picture of the experimental layout and greenhouse. (**C**) A graphical representation, and (**D**) a picture of the sensors used at each plot: I) a thermocouple, II) a quantum sensor, III) two soil moisture sensors, and IV) two load cells.

**Solar and LED spectrums by wavelength:**


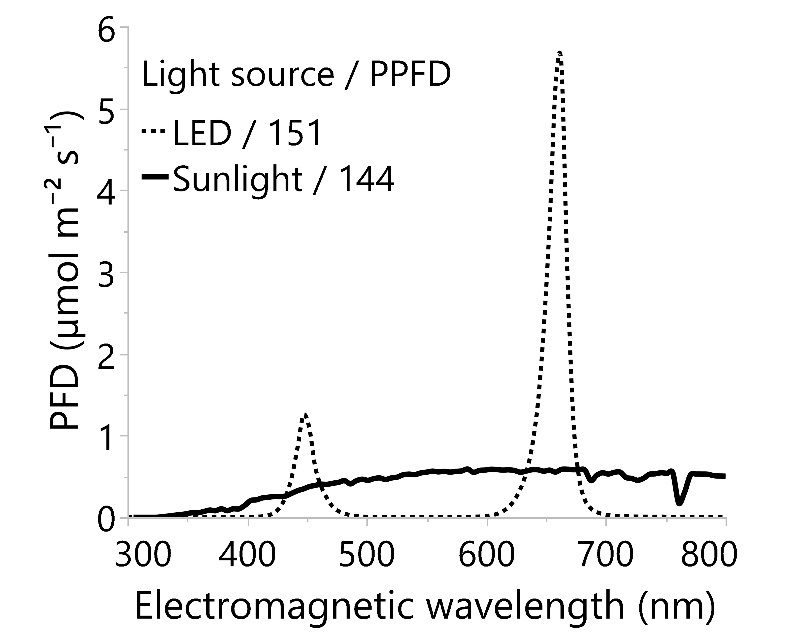


Figure S2. Average photon flux density (PFD) by nanometer of the electromagnetic spectrum between 300 and 800 nm from supplemental LED lighting (dotted line) and early in the afternoon clear sky sunlight (solid line) in the greenhouse. For comparison purposes, sunlight intensity was adjusted to align with an average PPFD of 144 µmol m^-2^ s^-1^, based on the LED photoperiod of 18 hours and the average solar daily light integral of 9.3 mol m^-2^ d^-1^.

**Supplemental light calibration and control:** The supplemental light levels were measured horizontally and vertically for calibration using a Li-191R line quantum sensor connected to a Li-1500 (Li-Cor, Lincoln, NE, USA). The line quantum sensor measured the entire length of the treatment area, including measurements on and between plant locations for the area width. To maintain constant light levels at the top of the crop canopy, the LED light output of each plot was digitally reduced based on the plot's average plant height every 2 to 4 days. To accomplish this, linear equations between the target light level and height were fitted for each plot before the experiment (R^2^ 0.991). Errors associated with the LED light distribution and dimming control were small (2.6 ± 1.7%, average light distribution deviation ± SD, and ≤ 5 µmol m^-2^ s^-1^, respectively).

The Li-191R line sensor was a brand-new sensor adapted to measure 91.5 cm (treatment area length) instead of 100 cm. Corroboration measurements were taken against a Li-190R and a PS-200 spectroradiometer (Apogee, Logan, UT, USA) in an open field. No errors from the quantum line sensor were observed.

**Irrigation and pot nutrient solutions:**

Table S1: pour-thru (LeBude and Bilderback, 2009) and irrigation nutrient solution analyses. The pour-thru samples among treatments were combined for nutrient analysis; pH and EC differences due to treatment effects were not observed before combining the samples. EC = electric conductivity. Tap water (pH 7.2, EC 0.2 dS m^-1^) was used biweekly and followed immediately by a nutrient solution cycle.

| **Solution source** | **Sampling date** | **pH** | **EC** | **N (NH4)** | **N (NO3)** | **P** | **K** | **Ca** | **Mg** | **S** | **Fe** | **Mn** | **Zn** | **Cu** | **B** | **Mo** | **Na** | **Cl** | **Alkalinity  (CaCO_3_)** |
| --- | --- | --- | --- | --- | --- | --- | --- | --- | --- | --- | --- | --- | --- | --- | --- | --- | --- | --- | --- |
| **Irrigation** | 10/09/20 | 6.1 | 1.9 | 17 | 177 | 42 | 185 | 93 | 42 | 14 | 5 | 0.71 | 0.45 | 0.07 | 0.97 | 0.07 | 29 | 14 | 21 |
|  | 10/24/20 | 6.2 | 1.8 | 16 | 169 | 45 | 173 | 93 | 41 | 16 | 5 | 0.70 | 0.40 | 0.06 | 1.01 | 0.07 | 29 | 14 | 21 |
| **Pour thru** (before Irrigation) | 10/24/20 | 5.9 | 2.3 | 2 | 200 | 38 | 147 | 188 | 61 | 81 | 4 | 0.15 | 1.20 | 0.33 | 0.27 | 0.00 | 40 | 20 | 24 |
| **Pour thru** (after Irrigation) | 11/02/20 | 6.3 | 1.9 | 1 | 170 | 50 | 131 | 124 | 42 | 35 | 7 | 0.04 | 1.32 | 0.16 | 0.45 | 0.00 | 61 | 27 | 34 |
| Units: |  |  | dS  m^-1^ | ppm | | | | | | | | | | | | | | | |

**Plot sensing and controlling hardware specifications:** Current Lighting Solutions (Greenville, SC, USA) provided the LED lights model L1000PPB. Soil moisture sensors, model ECH 10HS, were supplied by Meter Group (Pullman, WA, USA). Load cells, model RL1040-30KG, were from Rice Lake W.S. (Rice Lake, WI, USA). Fine thermocouples, model 5TC-TT-T-36-36, were from Omega (Norwalk, CT, USA). SQ-500 quantum sensors were provided by Apogee (Logan, UT, USA). The data acquisition and controlling system from Campbell Scientific devices (Logan, UT, USA) included a datalogger and controller (model CR1000), four multiplexers (model AM16/32), and a relay driver (model SDM16AC/DC).

**Substrate information and moisture calibration:** The commercial substrate mix utilized in this study (Pro-Line C/P, Jolly Gardener) has been detailed by Noah et al. (2022). A container with a substrate volume of 9.6 L from Elite Nursery Containers, model Classic 1200, with a top diameter of 10.5 inches and a height of 10 inches, was employed. All pots were filled with the same substrate fresh mass (3500 ± 1 g). Additionally, three extra pots were subjected to drying at 69°C for ten days to determine substrate dry mass (1.988 ± 0.056 kg, mean ± SD) and to calibrate the 10HS soil moisture sensors according to our experimental conditions. For calibration, known quantities of the irrigation solution were uniformly and cumulatively added, and voltage readings from multiple sensors were recorded in millivolts (mV). The volumetric water content equation, 6e^-07^*mV^2^ - 0.000123*mV - 0.0303, was used to control the irrigation in every plot. Throughout the experiment and calibration processes, the sensors were vertically inserted into the media with their tips positioned at 17.2 cm from the surface (sensor length 16.3 cm) and placed between the pot center and the wall. The larger sensing area of the 10HS sensors was oriented to face the center of the pots (for further details, see METER Environment).

**Load cells calibration, readings, and measurements:** Load cells were calibrated using precision weights (Troemner, Class U, NJ, USA). Evaporated and transpired water was calculated from the cumulative differences between measurements after the excess water from irrigation drained and just before the next irrigation cycle. The small plant mass increment and the water used by the plants around the irrigation cycle were not considered. See Figure S3a for more details.

Figure S3. (**A**) Load cell measurements for evaporation and evapotranspiration around an irrigation run time after 15 and 16 days in the greenhouse, sunny and cloudy days, respectively. Minimum and maximum values represent measurements to calculate water loss from evaporation and evapotranspiration. (**B**) Supplemental plus solar radiation PPFD corresponds with the plant-pot evapotranspiration. Measurements are plotted every 5 minutes.

**AI use:** ChatGPT 3.5 was used for language editing, e.g., prompt: “Please revise the grammar of the following text and show changes in bold: [text]”.

**RESULTS**

**Supplemental lighting effects on cutting production economic feasibility**

Given disease pressures and challenges with rooting cuttings in older plants (personal communication with nurseries), cannabis is frequently a seasonal crop in plant nurseries. For this reason, it is crucial for growers to have a dependable and expedited source of cuttings and cloned plants to enable year-round production and reduce order lead times. Consequently, the development of branches represents a critical morphological response for cannabis nursery production, with each branch offering the potential for conversion into a rooted cutting, thereby creating economic value. For instance, with a cost of approximately $2 per cutting (personal communication with a California nursery), an average U.S. electricity cost of $0.11 kWh, a light fixture efficiency of 3.1 μmol J^-1^ or higher (Current, 2023), and employing Huber et al. (2021) methodology and our findings of 0.41 branch mol^-1^ m^-2^, growers could make a revenue of $0.82 (0.41 cuttings * $2 per cutting) from the extra cutting production between 18 and 52 mol m^-2^ d^-1^, while spending on electricity about $0.01 per mole of supplemental light. It is important to note that the rate of 0.41 branch mol^-1^ m^-2^ will diminish while moving further out of this study’s DLI range. Moreover, the branch per mol rate can vary depending on the cultivar; as a reference, research by Suchoff et al. (2021) suggests that 'Suver Haze' is an average-yielding *Cannabis sativa* cultivar. Consequently, the rate of branches per mole of light could still be lower or higher than the one reported here. Nonetheless, based on this study’s findings and growing conditions, as well as the current price per cutting, increasing light intensity would not result in diminishing returns for cutting production.

**Supplemental lighting effects on internal carbon dioxide (C_i_) and leaf temperature**

Figure S4. (**A**) Leaf internal carbon dioxide concentration and (**B**) leaf and air temperatures from 150 to 2000 µmol m⁻² s⁻¹ (PPFD). The leaf chamber CO_2_ was maintained at greenhouse levels of 404 (µmol mol^-1^). Error bars represent the standard errors.

**REFERENCES**

Huber, B. M., Louws, F. J., and Hernández, R. (2021). Impact of Different Daily Light Integrals and Carbon Dioxide Concentrations on the Growth, Morphology, and Production Efficiency of Tomato Seedlings. Front. Plant Sci. 12, 615853. doi: 10.3389/fpls.2021.615853

LeBude, A.V., and Bilderback T.E. (2009). The Pour-Through Extraction Procedure: A Nutrient Management Tool for Nursery Crops. North Carolina Cooperative Extension, 1–8. Available at: <https://content.ces.ncsu.edu/the-pour-through-extraction-procedure-a-nutrient-management-tool-for-nursery-crops>.

Suchoff, D., Bloomquist, M., Davis, J., Henriquez Inoa, S., Learn K. (2021). NC State Floral Hemp Variety Trial. North Carolina Extension 2021. Available at: https://hemp.ces.ncsu.edu/wp-content/uploads/2021/11/2021-NC-State-Variety-Trial-Report.pdf?fwd=no [Accessed January 7, 2024].
